# Mevalonate production from ethanol by direct conversion through acetyl-CoA using recombinant *Pseudomonas putida*, a novel biocatalyst for terpenoid production

**DOI:** 10.1186/s12934-019-1213-y

**Published:** 2019-10-10

**Authors:** Jeongmo Yang, Ji Hee Son, Hyeonsoo Kim, Sukhyeong Cho, Jeong-geol Na, Young Joo Yeon, Jinwon Lee

**Affiliations:** 1 Department of Chemical and Biomolecular Engineering, Sogang University, Seoul, 04107 Republic of Korea; 2 C1 Gas Refinery R&D Center, Sogang University, Seoul, 04107 Republic of Korea; 3 Department of Biochemical Engineering, Gangneung-Wonju National University, Gangneung, 25457 Republic of Korea

**Keywords:** Mevalonate, Ethanol, *Pseudomonas putida*

## Abstract

**Background:**

Bioethanol is one of the most representative eco-friendly fuels developed to replace the non-renewable fossil fuels and is the most successful commercially available bio-conversion technology till date. With the availability of inexpensive carbon sources, such as cellulosic biomass, bioethanol production has become cheaper and easier to perform, which can facilitate the development of methods for converting ethanol into higher value-added biochemicals. In this study, a bioconversion process using *Pseudomonas putida* as a biocatalyst was established, wherein ethanol was converted to mevalonate. Since ethanol can be converted directly to acetyl-CoA, bypassing its conversion to pyruvate, there is a possibility that ethanol can be converted to mevalonate without producing pyruvate-derived by-products. Furthermore, *P. putida* seems to be highly resistant to the toxicity caused by terpenoids, and thus can be useful in conducting terpenoid production research.

**Results:**

In this study, we first expressed the core genes responsible for mevalonate production (*atoB*, *mvaS*, and *mvaE*) in *P. putida* and mevalonate production was confirmed. Thereafter, through an improvement in genetic stability and ethanol metabolism manipulation, mevalonate production was enhanced up to 2.39-fold (1.70 g/L vs. 4.07 g/L) from 200 mM ethanol with an enhancement in reproducibility of mevalonate production. Following this, the metabolic characteristics related to ethanol catabolism and mevalonate production were revealed by manipulations to reduce fatty acid biosynthesis and optimize pH by batch fermentation. Finally, we reached a product yield of 0.41 g mevalonate/g ethanol in flask scale culture and 0.32 g mevalonate/g ethanol in batch fermentation. This is the highest experimental yield obtained from using carbon sources other than carbohydrates till date and it is expected that further improvements will be made through the development of fermentation methods.

**Conclusion:**

*Pseudomonas putida* was investigated as a biocatalyst that can efficiently convert ethanol to mevalonate, the major precursor for terpenoid production, and this research is expected to open new avenues for the production of terpenoids using microorganisms that have not yet reached the stage of mass production.

## Background

Bioethanol has been one of the most prominent and eco-friendly alternatives to fossil fuels, which has been used in ground transportation. By 2020, all the countries in the European Union (EU) will be required to raise their renewable energy share by 20% and the development of bio-ethanol production technology is becoming more important. Carbohydrates from grains are the major feedstocks for bioethanol production, but this causes food-fuel competition [1]. Therefore, utilization of the commonly available lignocellulosic biomass or even waste as carbon source for bioethanol production is one way to resolve this competition [2]. Furthermore, an alternative new generation of bioethanol production process which uses syngas (produced from gasification of biomass) or blast furnace gas (by-product of the steel industry) composed of CO, CO_2_, and H_2_, has come to the forefront [3]. Acetogen, mainly belonging to the genus *Clostridium,* can produce ethanol from CO, CO_2_, and H_2_ through the Wood–Ljungdahl pathway, also known as the reductive acetyl-coenzyme A (acetyl-CoA) pathway [4]. With the availability of these less expensive carbon sources, bioethanol production process will be cheaper and more abundant, which can be a motivation for newer, innovative process development to convert ethanol into higher value-added biochemicals.

Since ethanol can be converted to mevalonate without any carbon loss, we have selected mevalonate to be the final product. Mevalonate can be used as a moisturizer in cosmetics, a monomer of a new class of polyesters with tunable mechanical properties through a simple chemical reaction to β-methyl-δ-valerolactone [5], and a key metabolic precursor for the biosynthesis of terpenoids, including isoprene, limonene, and farnesene, which are widely used as monomers, biofuels, and cosmetics [6]. Until now, mevalonate or terpenoid production studies have focused mainly on the well-characterized species, such as *Escherichia coli* and *Saccharomyces cerevisiae*, but our study for the first time shows *Pseudomonas putida* as a novel and effective mevalonate producer. *P. putida,* a gram-negative soil bacterium, is known to utilize not only the aromatic hydrocarbons but also various alcohols, naturally, as a carbon source in aerobic conditions [7, 8]. Furthermore, *P. putida* seems to be highly resistant to terpenoids, similar to the mechanism of non-polar hydrocarbons, and this characteristic is often considered to be advantageous in further production of terpenoid from mevalonate [9].

**Fig. 1:**
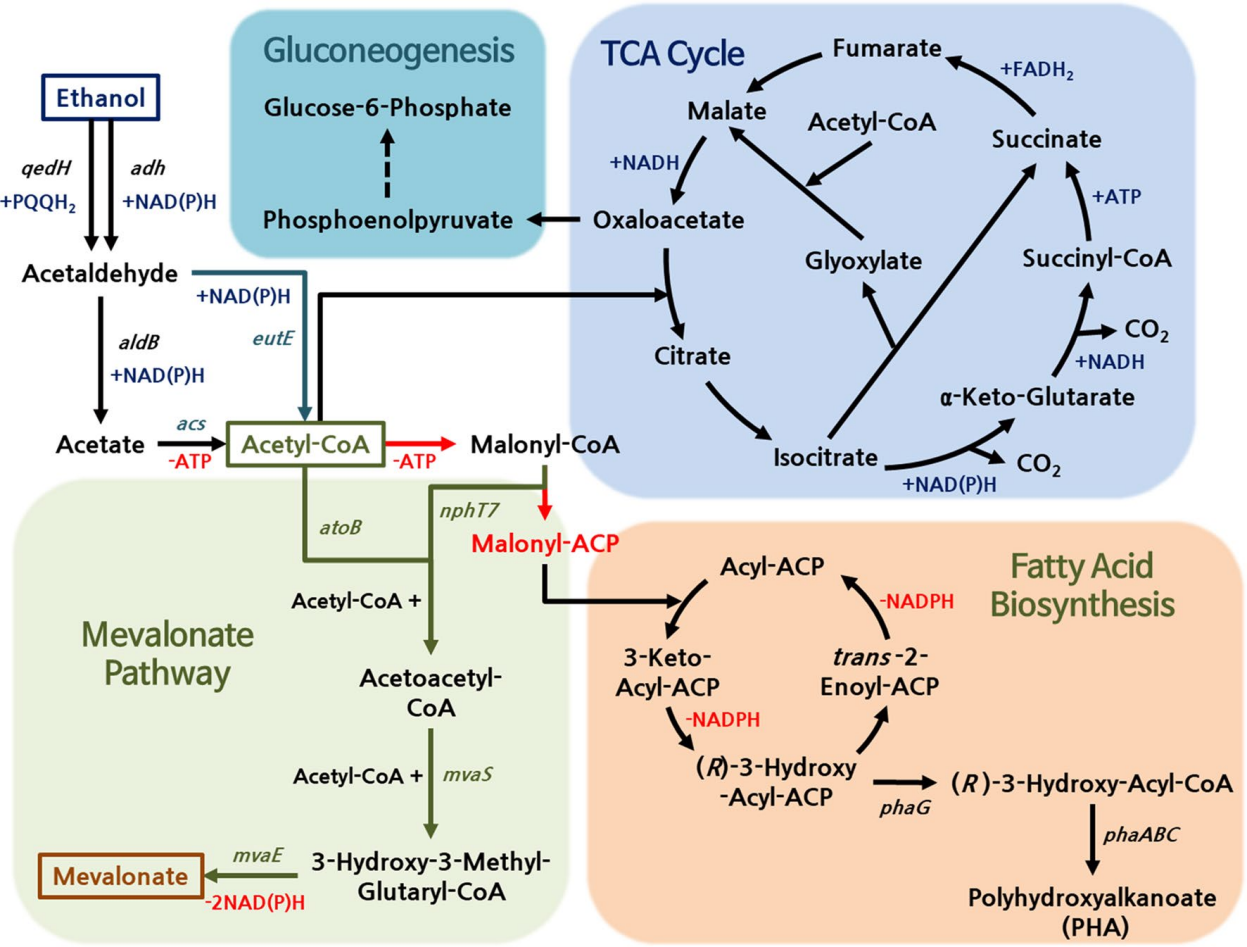
Metabolic pathway from ethanol to mevalonate in *P. putida* KT2440

In *P. putida*, ethanol is oxidized to acetaldehyde in two ways, one is periplasmic oxidation of ethanol using pyrroloquinoline quinone (PQQ) as a cofactor in the aerobic conditions and the second is the cytosolic oxidation of various alcohol using an oxidized NAD or NADP as a cofactor. Acetaldehyde, a toxic chemical, produced from the two types of oxidation, may be converted to acetyl-CoA via acetate by the *aldB* and *acs* genes (encoding acetaldehyde dehydrogenase B and acetyl-CoA synthetase, respectively) with the generation of one molecule of NADH and consumption of one molecule of ATP. In summary, ethanol can be converted to acetyl-CoA with the generation of NADH without any carbon loss (non-CO_2_ generation) in *P. putid*a KT2440 [10]. The acetyl-CoA generated from ethanol catabolism is directly introduced into the heterologous mevalonate pathway for the synthesis of mevalonate, a precursor of terpenoid (acetyl-CoA to mevalonate conversion step). Terpenoids are synthesized via two modules of the biosynthetic pathway, which are the mevalonate pathway and the 2-C-methyl-d-erythritol 4-phosphate/14-deoxy-d-xylulose 5-phosphate (MEP/DOXP) pathway. The mevalonate pathway, the eukaryotic module of terpenoid backbone biosynthesis, produces one C5 isoprene monomer (DMAPP or IPP) from three molecules of acetyl-CoA. The MEP/DOXP pathway is the bacterial module of terpenoid backbone biosynthesis, which produces one DMAPP or IPP from two molecules of pyruvate. Generally, in the bacterial production of terpenoids, the heterologous mevalonate pathway is often expressed rather than the MEP/DOXP pathway due to the complicated feedback control mechanisms and pathway configurations [9, 11]. When ethanol is used as a carbon source, unlike glucose or glycerol, it is converted directly into acetyl-CoA, without carbon loss to carbon dioxide, while supplying reducing power for mevalonate biosynthesis sufficiently. Additionally, since ethanol is converted to acetyl-CoA bypassing pyruvate, this conversion has an advantage of producing no by-products derived from pyruvate. The metabolic pathway from ethanol to mevalonate is shown in Fig. 1.

In this study, a bioconversion process for creating mevalonate from ethanol using *P. putida* as a biocatalyst has been established. First, the upper mevalonate pathway was constructed in *P. putida* through the heterologous expression of codon-optimized *mvaE* (acetyl-CoA acetyltransferase/hydroxymethylglutaryl-CoA reductase from *Enterococcus faecalis*), *mvaS* (hydroxymethylglutaryl-CoA synthase from *Enterococcus faecalis*), and *atoB* (acetyl-CoA acetyltransferase from *Escherichia coli*) genes and mevalonate production was confirmed. Second, to overcome the instability and low efficiency of mevalonate production, genetic stability was improved by the deletion of endonuclease genes (*endA* and *endX*) and ethanol catabolism was manipulated to prevent acetate accumulation, the inhibitory factor for mevalonate production. Third, to enhance the mevalonate production, fatty acid biosynthesis, a major bypath of acetyl-CoA flux, was hindered by introducing an alternative step of acetoacetyl-CoA synthesis and blocking the polyhydroxyalkanoate (PHA) synthesis pathway.

## Results

### Mevalonate production from ethanol

**Fig. 2:**
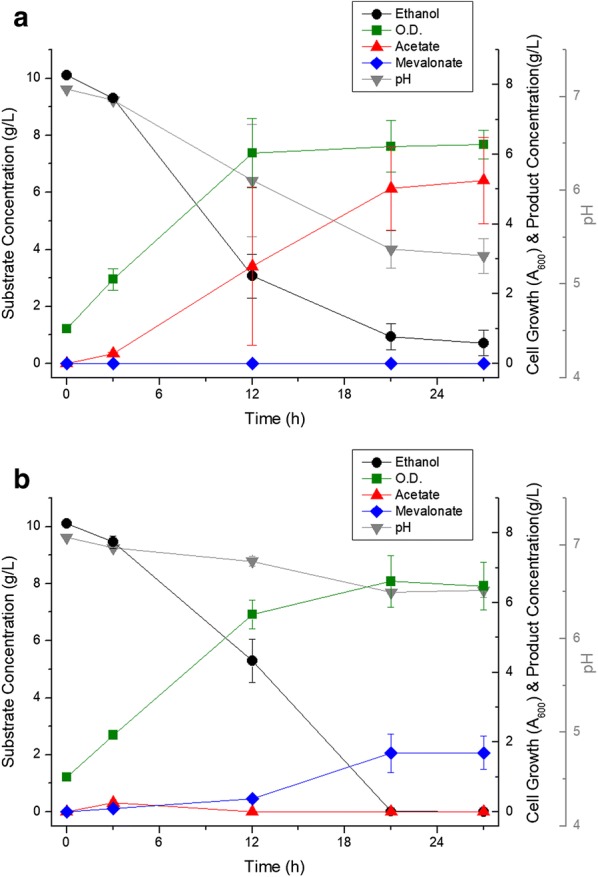
Metabolic profile of **a** ELPP000 and **b** ELPP010 in M9 ethanol media. Black circles indicate ethanol concentration, green squares indicate cell growth (optical density at 600 nm), red triangles indicate acetate concentration, blue diamonds indicate mevalonate concentration, and gray inverted triangles indicate pH of media. All the experiments were performed in triplicates and standard deviations of triplet culture were shown in the form of error bars

Flask scale fermentation was carried out to confirm that mevalonate was produced when the *atoB*, *mvaS*, and *mvaE* genes (upper mevalonate pathway) were preferentially expressed. The metabolic profiles of ELPP000 and ELPP010 strains are illustrated in Fig. 2. In the ELPP000 strain, which acts as a negative control, ethanol was rapidly metabolized during the initial 12 h of fermentation, but cell growth was inhibited only after 12 h due to a decrease in pH by the acetic acid (a metabolic intermediate) accumulation. Finally, a large amount of acetate was not metabolized and thus it accumulated. However, the ELPP010 strain, into which the upper mevalonate pathway was introduced, produced 1.70 ± 0.55 g/L of mevalonate after 27 h, mainly after 12 h of aerobic ethanol metabolism. Unlike ELPP000, no acetate was accumulated, and all the ethanol was used for cell growth and mevalonate production. The pH after fermentation was higher than that of ELPP000. Due to the difference in the molar ratio between the acetate and mevalonate produced from ethanol, the mevalonate production has a relatively lower pH change than acetate accumulation. Although mevalonate was produced, the production yield (Y_P/S_) was 0.168, which was much lower than the theoretical yield of 1.07. When calculating the production yield, carbon sources other than ethanol were neglected.

### Genetic stability improvement in *P. putida* KT2440

**Fig. 3:**
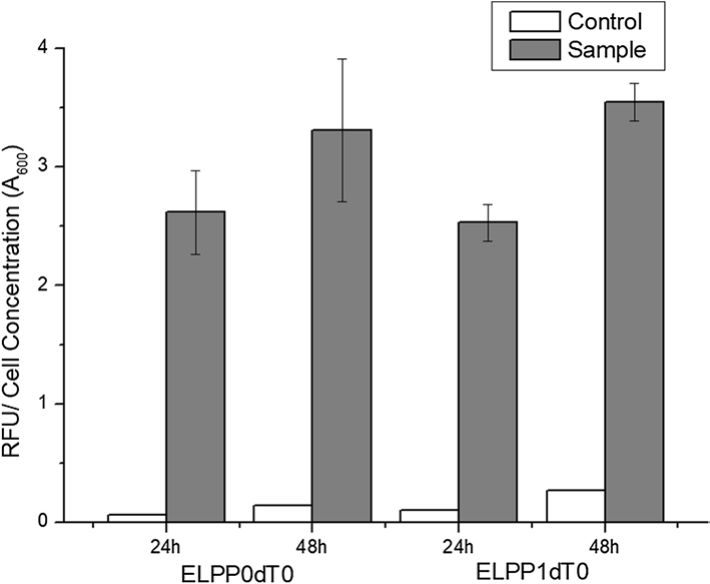
Fluorometric quantification of dTomato protein for ensuring genetic stability improvement. White bars indicate the control value (did not induced by IPTG) and gray bars indicate the sample value. All the experiments were performed in triplicates and standard deviations of triplet culture were shown in the form of error bars

In order to increase the yield of mevalonate, we focused on the genetic stability of *P. putida*, which has various defense mechanisms to defend against invasion of heterologous DNA. Due to this defense mechanism, the expression level of the foreign gene is decreased as the fermentation time passes by and with each succeeding generation, the heterologous plasmid vector decomposes or is discharged from the cells. In this study, *endA* and *endX* genes encoding endonuclease, involved in the mechanism of foreign DNA degradation were deleted from the chromosomal DNA of *P. putida* KT2440. To confirm the improvement of genetic stability, wild-type and mutant strain harboring pSGP10 vector cloned with dTomato, a red fluorescence protein, were cultured in M9 minimal media and the cell fluorescence intensity (excitation 544 nm, emission 590 nm) was estimated using a microplate fluorescence reader. In endonuclease deleted mutant, the increase in relative fluorescence signal with incubation time was more than the control strain (1.27-fold increase in ELPP0dT0 and 1.40-fold increase in ELPP1dT0 from 24 to 48 h) and this indicated that the stability of the heterologous vector was improved in the mutant strain. In addition, the standard deviation of triplet experiments was reduced in the mutant strain as compared to the control (3.74-fold decrease in ELPP1dT0 at 48 h), indicating that the stability of the gene expression was also improved (Fig. 3).

**Fig. 4:**
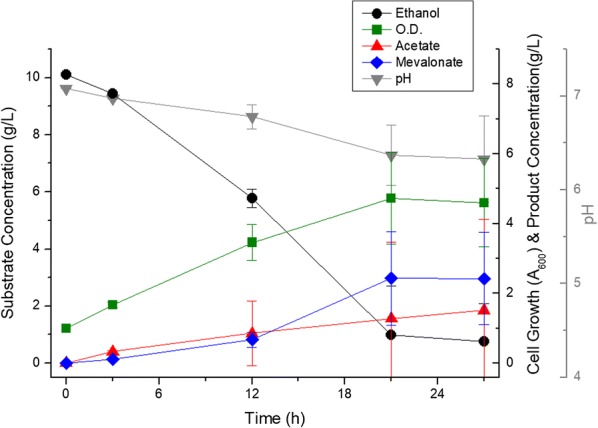
Metabolic profile of ELPP110 in flask scale. Black circles indicate ethanol concentration, green squares indicate cell growth (optical density at 600 nm), red triangles indicate acetate concentration, blue diamonds indicate mevalonate concentration, and gray inverted triangles indicate pH of media. All the experiments were performed in triplicates and standard deviations of triplet culture were shown in the form of error bars

After fluorescence analysis, 2.43 ± 1.34 g/L of mevalonate was produced by a 27 h flask scale fermentation of the ELPP110 strain (Fig. 4). In comparison to ELPP010, it showed a remarkable increase of about 43%, and it was thus confirmed that the improvement in the stability of foreign gene is an important factor that can continuously supply the acetyl-CoA to mevalonate production. However, the reproducibility of mevalonate production in the triplicate experiment were greatly decreased. In some flasks, the cell growth and mevalonate production were abruptly inhibited by the decrease in pH due to the rapid accumulation of acetate and in the other flasks the mevalonate was successfully produced without acetate accumulation. From this result, it was found that preventing accumulation of acetate would be another important factor for successful mevalonate production.

### Manipulation of ethanol catabolism

**Fig. 5:**
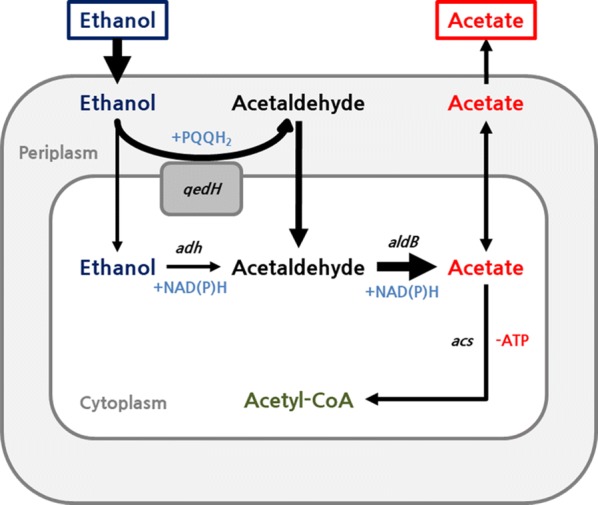
Schematic diagram of ethanol catabolism in *P. putida* KT2440

**Fig. 6:**
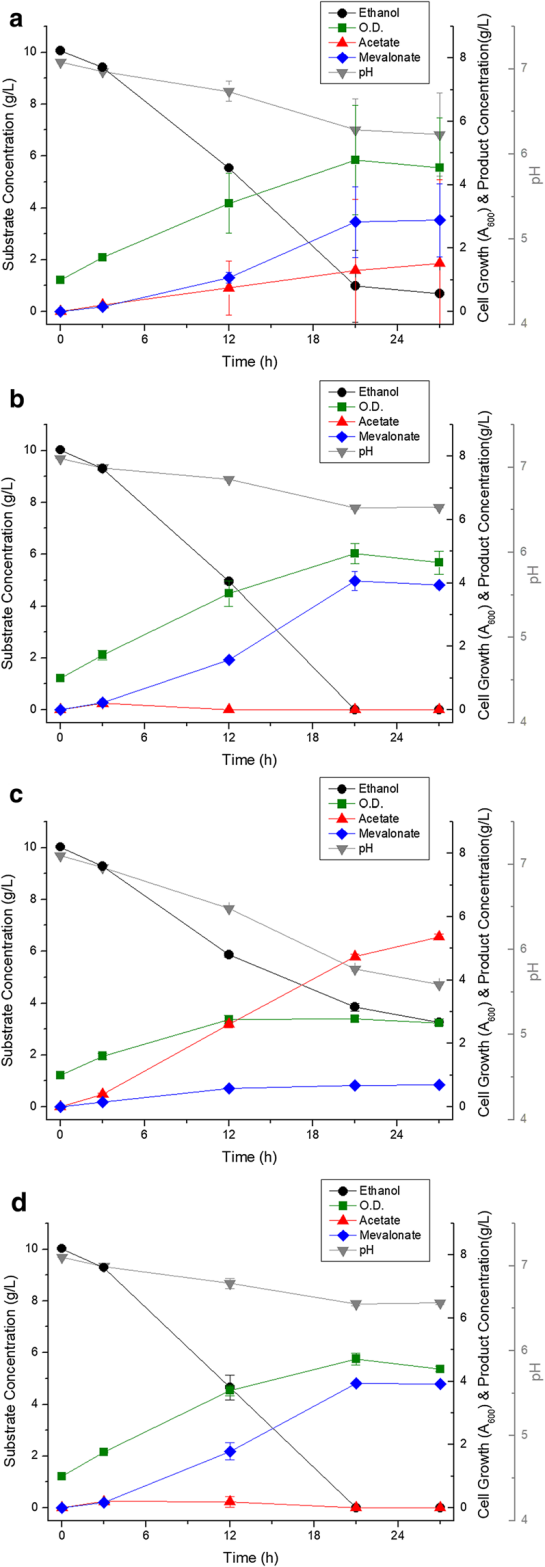
Metabolic profile of **a** ELLPP111, **b** ELPP211, **c** ELPP212 and **d** ELPP213 in flask scale Black circles indicate ethanol concentration, green squares indicate cell growth (optical density at 600 nm), red triangles indicate acetate concentration, blue diamonds indicate mevalonate concentration, and gray inverted triangles indicate pH of media. All the experiments were performed in triplicates and standard deviations of triplet culture were shown in the form of error bars

Although improved genetic stability greatly enhanced the yield of mevalonate, the fermentation reproducibility was decreased by acetate accumulation. A summary of ethanol catabolism in *P. putida* is shown in Fig. 5. To solve the accumulation of acetate, recombinant DNA technology was used. First, *acs* (acetyl-CoA synthetase) derived from *Escherichia coli* MG1655, which is a gene involved in introducing acetate into the cellular metabolism, was expressed. ELPP111, *acs* gene expressed in ELPP110, produced 2.88 ± 1.16 g/L of mevalonate in 27 h flask fermentation and it showed a slight increase in mevalonate production but not in reproducibility (Fig. 6a). Quinoprotein ethanol dehydrogenase (coded by *qedH*-*I* and *II* genes), a membrane-bound protein, also acts as an enzyme responsible for the periplasmic oxidation of ethanol to acetaldehyde in the aerobic conditions of *P. putida*, with a high conversion efficiency [12]. We hypothesized that the observed decrease in reproducibility is caused by the imbalance between the rate of oxidation process of ethanol to acetate facilitated by quinoprotein ethanol dehydrogenase and the rate of acetate conversion to acetyl-CoA and further introduction into cellular metabolism. While the rate of oxidation of ethanol to acetate is very fast, the rate of conversion of acetate to acetyl-CoA is limited by the cell condition and this imbalance could cause the acetate accumulation by insignificant transition in the culture condition. Thus, removal of *qedH* gene would reduce the ethanol catabolism rate, with an assumption that there would be no significant effect on ethanol metabolism due to the presence of various alcohol dehydrogenases in *P. putida.* ELPP211, *qedH*-*I* and *qedH*-*II* deleted strain from ELPP111, produced 4.07 ± 0.29 g/L of mevalonate without acetate accumulation and showed a remarkable improvement in reproducibility compared to the ELPP111 strain (Fig. 6b). Furthermore, in order to increase the efficiency of ethanol conversion into acetyl-CoA, expression of *eutE* gene (coding putative aldehyde dehydrogenase), which can synthesize acetyl-CoA from acetaldehyde without consuming ATP, was established. The ELPP212 strain, *eutE* expressed without *acs,* showed negative effect on mevalonate production, and there was no improvement as compared to ELPP211 in the ELPP213 strain, *eutE* and *acs* co-expressed (Fig. 6c, d).

### Reduction of acetyl-CoA flux into fatty acid biosynthesis

**Fig. 7:**
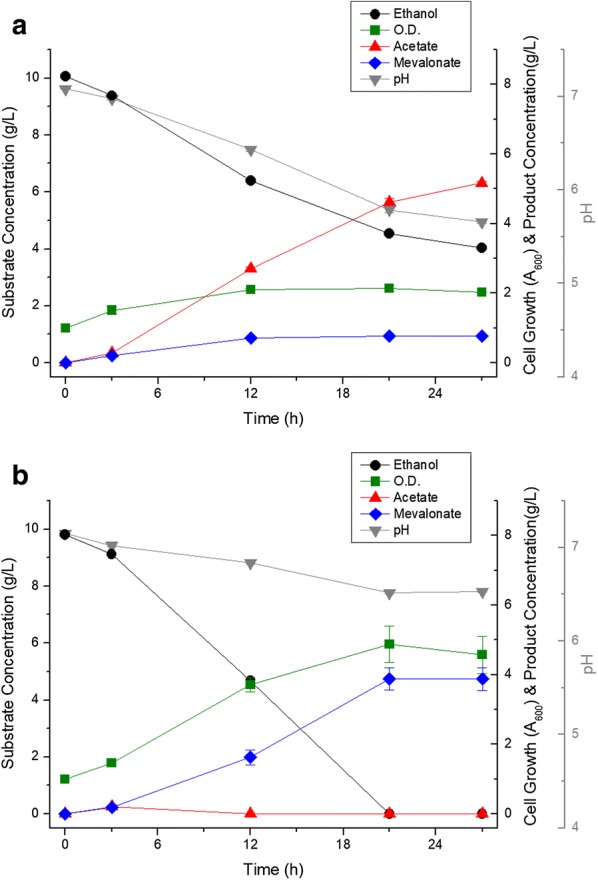
Metabolic profile of **a** ELPP221 and **b** ELPP311 in flaks scale. Black circles indicate ethanol concentration, green squares indicate cell growth (optical density at 600 nm), red triangles indicate acetate concentration, blue diamonds indicate mevalonate concentration, and gray inverted triangles indicate pH of media. All the experiments were performed in triplicates and standard deviations of triplet culture were shown in the form of error bars

**Fig. 8:**
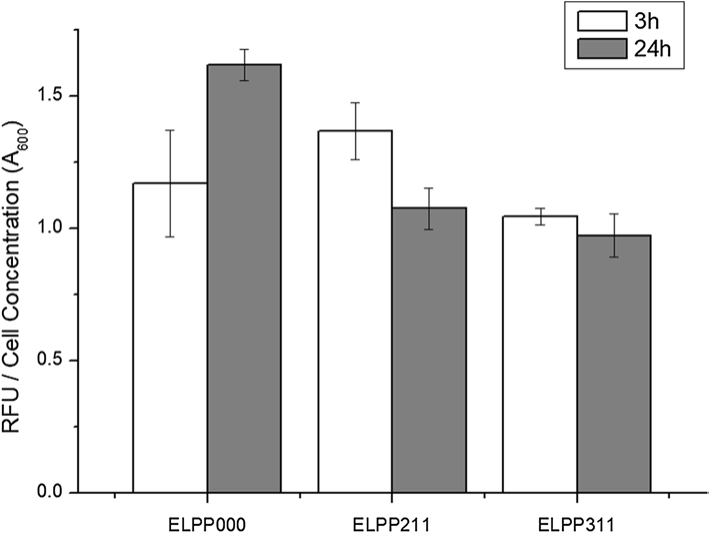
Fluorometric quantification of neutral lipids using Nile red in ELPP000, ELPP211 and ELPP311. White bars indicate the value of 3 h culture time and gray bars indicate the value of 24 h culture time. All the experiments were performed in triplicates and standard deviations of triplet culture were shown in the form of error bars

Fatty acids, synthesized from acetyl-CoA, are one of the major by-products of mevalonate production. Especially, it is known that *P. putida* synthesizes and accumulates polyhydroxyalkanoates (PHA) from almost all carbon sources [13]. In this study, two strategies for inducing acetyl-CoA flux to mevalonate by inhibition of fatty acid production were designed. First, acetoacetyl-CoA synthase (encoded by *nphT7* from *Streptomyces* sp.), which synthesizes acetoacetyl-CoA from acetyl-CoA and malonyl-CoA, was expressed in order to introduce malonyl-CoA (synthesized from acetyl-CoA and serve as a precursor of fatty acid biosynthesis) into the mevalonate pathway [14]. However, the ELPP221 strain, *nphT7* expressed in ELPP211, showed very strong cell growth inhibition (Fig. 7a). In order to inhibit the synthesis of PHA which is surplus carbon storage source, without cell growth inhibition, the *phaG* gene, a branch point into PHA biosynthesis from the fatty acid elongation step, was deleted. In the ELPP311 strain, *phaG* deleted from ELPP211, there was no difference in the production of mevalonate from the ELPP211 strain (Fig. 7b). To demonstrate the absence of *phaG* gene deletion effect, the total amount of neutral fatty acids in the ELPP000 strain which did not have the mevalonate pathway, mevalonate-producing strain ELPP211, and the *phaG* gene deleted ELPP311 strain, was analyzed by Nile red staining fluorescence analysis. After 3 h of cultivation, the neutral fatty acid content of ELPP211 was the highest, but only the wild-type ELPP000 increased the neutral fatty acid contents and that of ELPP211 and ELPP311 strains decreased after consumption of all the ethanol (Fig. 8). The fact that both ELPP211 and ELPP311 strains reduced the neutral fatty acid content suggests that the construction of the mevalonate pathway can prevent the conversion of excess acetyl-CoA to PHA, which would in turn explain the reason behind no increase in mevalonate production when the *phaG* gene is deleted.

### Batch fermentation—pH effect

**Fig. 9:**
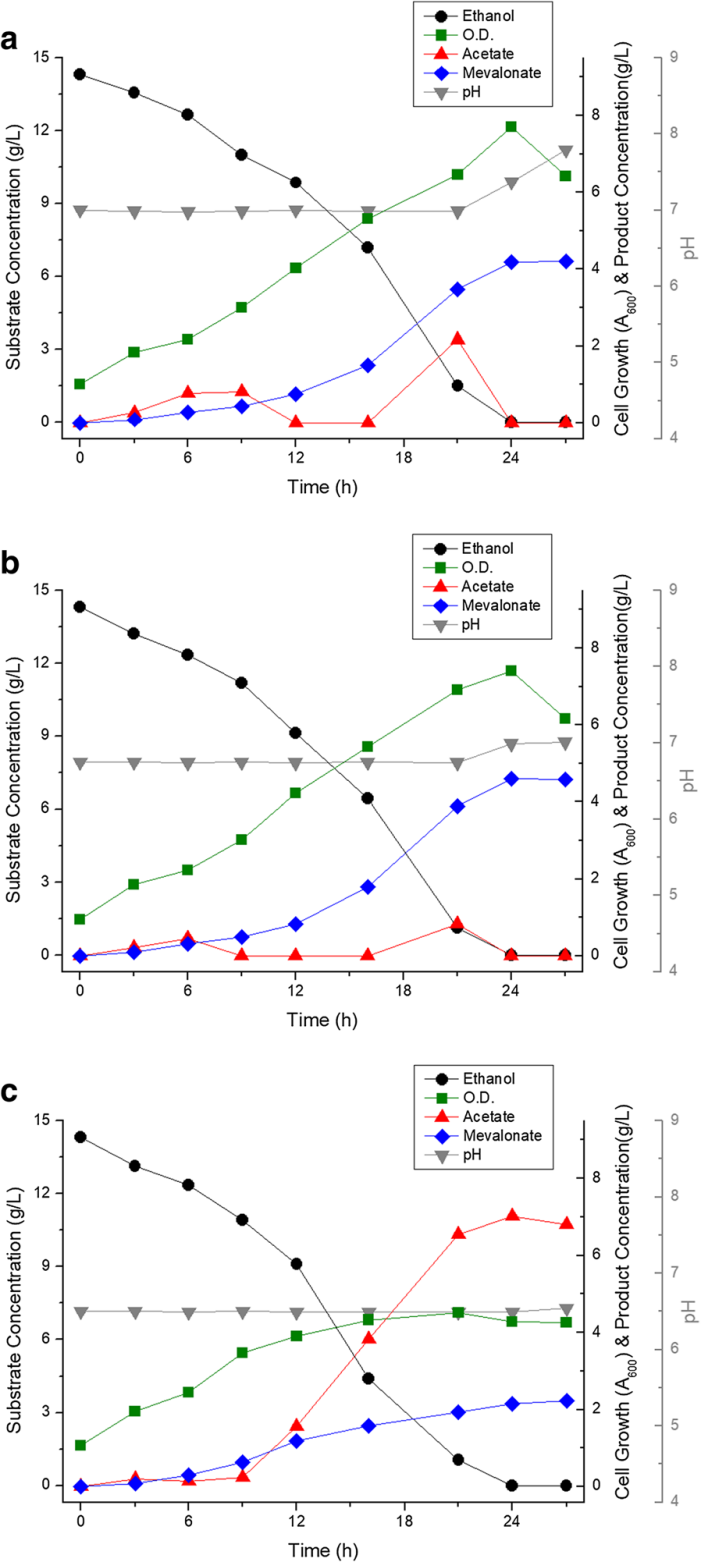
Metabolic profile of ELPP311 in batch fermenter at **a** pH 7.0, **b** pH 6.75 and **c** pH 6.5 control. Black circles indicate ethanol concentration, green squares indicate cell growth (optical density at 600 nm), red triangles indicate acetate concentration, blue diamonds indicate mevalonate concentration, and gray inverted triangles indicate pH of media

After strain selection from flask scale culture, 1 L scale batch fermentation was performed. The difference in batch fermentation from flask culture is that the pH can be controlled, and batch fermentation can indicate the tendency of microorganism growth and metabolite production depending on pH. Basic fermentation was carried out under the condition of pH 7.0, which is known to be the most suitable for *P. putida* growth, a neutrophilic microorganism. At pH 7.0 control culture, 4.18 g/L of mevalonate was produced from 13.6 g/L of ethanol in 24 h culture time, which was due to the reduction in the production yield compared to the flask culture (Fig. 9a). On the other hand, the growth of bacteria was significantly increased up to OD_600_ 7.7, with an accumulation of acetate which disappeared after 16 h of culture time. At pH 6.75 condition, the overall growth and appearance of mevalonate production was similar to that at pH 7.0, but the accumulation of intermediate acetate was reduced and the final yield slightly increased (Fig. 9b). The steps taken to increase the production yield was by preventing acetate accumulation and promoting cell overgrowth, was by decreasing the pH to 6.5, which was not a lethal condition for *P. putida* growth as it is a neutrophilic bacterium. However, at pH 6.5, the intermediate acetate accumulated and thus the rate of mevalonate production was greatly reduced after 12 h of culture time (Fig. 9c). From the result of pH optimization batch culture, it was concluded that a slightly acidic pH (about 6.75) could be optimal for the mevalonate production using control batch fermentation and that 4.60 g/L of mevalonate was produced from 14.3 g/L of ethanol (about 300 mM) with a production yield of 0.32 g mevalonate/g ethanol.

## Discussion

Ethanol is a carbon source that provides significant reducing power and is directly converted to acetyl-CoA, a key intermediate in cell metabolism. It can be used as a carbon source to obtain various biochemicals synthesized from acetyl-CoA, as a precursor, in high yield. Terpenoids are representative biochemical compounds synthesized from acetyl-CoA. *P. putida* is a strain which is highly resistant to the toxicity of terpenoids and is considered as a useful strain for producing terpenoids using microorganisms [9]. Despite these advantages, studies on the production of terpenoids from ethanol in *P. putida* have not been conducted so far, and only theoretical merits have been proposed. To demonstrate this advantage and to determine whether further research is worthwhile, the study has been conducted to convert ethanol to mevalonate, a key intermediate in terpenoid synthesis.

First, to confirm mevalonate production, the upper mevalonate pathway (from acetyl-CoA to mevalonate) was constructed in *P. putida*. The ELPP000 strain showed a typical form of aerobic metabolism, where the cells rapidly grew to certain cell concentration without any metabolite production and then they oxidized ethanol to acetate for energy supply without completely catabolizing the ethanol. However, the ELPP010 strain showed a facultative metabolism that converted acetyl-CoA (produced from ethanol) to mevalonate, even after cell growth through aerobic metabolism. This phenomenon was attributed to the fact that the acetyl-CoA can be consumed for CoA recycling by the metabolic pathway to the mevalonate, and that the reducing power, generated from ethanol oxidation to acetyl-CoA, was sufficient to be used for both cellular metabolism and mevalonate synthesis.

To enhance the mevalonate yield, the research focused on genetic stability of the plasmid vector. In wild-type *P. putida*, there are various defense mechanisms to prevent foreign gene invasion and internal DNA mutation [15]. Previous studies have clarified that the defense mechanism of *P. putida* reduced the efficiency of heterologous gene expression through the plasmid vector and eliminating these defense mechanisms improved the efficiency of heterologous gene expression through a series of experiments [16]. In this study, the endonucleases of *P. putida* were eliminated for enhancing the efficiency of heterologous gene expression among the various defense mechanisms. Fluorescence analysis through dTomato protein expression revealed that the stability of protein expression was slightly increased with incubation time, but the efficiency of mevalonate production increased remarkably. Endonuclease has the function of preventing foreign gene entry as well as correcting the mutations of internal DNA, removal of endonuclease delays cell growth due to the inability in efficient DNA repair [17], which also provides an environment that can efficiently induce acetyl-CoA to mevalonate synthesis, in addition to the stable expression of enzymes involved in mevalonate synthesis. Furthermore, the elimination of endonuclease caused an accumulation of acetate in some fermentations and resulted in an increase in the standard deviation of fermentation.

The accumulation of acetate can be attributed to the low expression level of acetyl-CoA synthetase that converts acetate to acetyl-CoA, or an imbalance in the rate at which ethanol is converted to acetate and the rate at which acetate is converted to acetyl-CoA. To elucidate the first cause, the *acs* gene derived from *E. coli* was expressed. Although acetate accumulation was relatively decreased, and the mevalonate production was increased, the reproducibility problem was not fundamentally solved. This suggested that it was necessary to slow the rate of oxidation of ethanol to acetate. Unlike *E. coli*, *Pseudomonas* has the PQQ dependent ethanol dehydrogenase (designated by *qedH*) that oxidizes ethanol very rapidly to acetaldehyde at the periplasm, which gives it the ability to grow rapidly by metabolizing ethanol [10]. However, the rapid oxidation rate of ethanol in periplasm could be attributed to the accumulation of acetate on the outside of the cell, which may rapidly decrease the pH and cause the cell to lose its activity. Therefore, the *qedH* gene responsible for periplasmic oxidation was removed in order to slow down the rapid oxidation of ethanol and prevent rapid accumulation of acetate. In general, the removal of *qedH* might slow the rate of ethanol oxidation, which in turn might lead to the inability to metabolize ethanol. However, in actual experiments, it was confirmed that ethanol could be metabolized in the absence of unnecessary accumulation of acetate. This could be presumably be due to the oxidation of ethanol by a wide variety of alcohol dehydrogenases in cytoplasm. In addition, *eutE* from *E. coli,* which directly converts acetaldehyde to acetyl-CoA, was expressed to reduce acetate production and ATP consumption. However, this resulted in an accumulation of acetate, which can be explained by the action of aldehyde dehydrogenase. The putative aldehyde dehydrogenase, coded by *eutE,* is a reversible enzyme that also converts acetyl-CoA to acetaldehyde and the chances of reverse reaction could be major because the concentration of toxic acetaldehyde in cytoplasm is very low in vivo.

In *P. putida* metabolism, acetyl-CoA is mainly used in the TCA cycle for energy production, gluconeogenesis for sugar-derived precursor synthesis, and intracellular fatty acid synthesis. All of these cannot be manipulated indiscreetly, because they are essential for cell growth. In order to direct acetyl-CoA to mevalonate synthesis exclusively, a method of expressing acetoacetyl-CoA synthase, which can synthesize acetoacetyl-CoA from acetyl-CoA and malonyl-CoA (a precursor of fatty acid biosynthesis), should be employed. However, unlike the expectation that high malonyl-CoA would be used for mevalonate synthesis, malonyl-CoA was found to be excessively used for mevalonate synthesis with a remarkable reduction in cell growth. The leakage of acetyl-CoA was blocked, and mevalonate production was increased by blocking the synthesis of PHA that stores extra carbon sources, rather than inducing malonyl-CoA to mevalonate. *P. putida* is known to store carbon sources in the form of PHA (a medium chain fatty acid polymer) in a specific condition. However, some groups have reported that a certain amount of PHA is synthesized and stored even in a normal growth environment [12]. Based on these results, it was determined that inhibition of PHA synthesis would increase mevalonate production without inhibiting growth. Deletion of *phaG* gene coding branch point into PHA synthesis from fatty acid elongation, could inhibit the PHA synthesis briefly [17]. As a result, the *phaG* deleted strain (ELPP311) did not produce further mevalonate as compared to the ELPP211 strain and it was confirmed that the ELPP211 strain did not accumulate PHA in the cells at a level similar to that of the ELPP311. Upper mevalonate pathway construction enabled the cell to induce mevalonate synthesis of all acetyl-CoA other than those necessary for cell survival.

In pH optimization batch fermentation, trends in ethanol metabolism, cell growth, and mevalonate production with pH was observed. Especially, the ethanol metabolism and mevalonate production decreased sharply under the condition at pH 6.5. In the case of fermentative neutrophilic bacteria such as *E. coli*, cell growth and metabolism are maintained even near the pH of about 5, *P. putida* being an obligate aerobe which cannot generate the proton motive force other than aerobic respiration, and this cell growth and metabolism may be affected more with changes in pH [18]. In fact, obligate aerobes such as *P. putida* and *B. subtilis*, compared to *E. coli*, have proven that the gradient of the change in intracellular ATP contents is higher with external pH changes [19]. At a slightly milder pH of 6.75, more effective mevalonate production without acetate accumulation was observed, and the maximum cell growth was reduced compared to pH 7.0, without any significant difference in the production rate. However, production yield in batch fermentation was decreased to 0.32 g mevalonate/g ethanol from 0.41 g mevalonate/g ethanol in flask fermentation. This decrease in production is thought to be due to volatilizing and escaping of ethanol because air is directly injected from the lower surface at a relatively fast rate in batch fermentation. In fact, it was directly confirmed that about 2 g/L of ethanol was lost in the uninoculated medium.

## Conclusions

In this study, the biocatalyst that can efficiently convert ethanol to mevalonate, the major precursor for terpenoid production, was developed. First, the core genes for mevalonate production (*atoB*, *mvaS*, *mvaE* gene) were expressed in *P. putida* and mevalonate production was confirmed. Second, through genetic stability improvement and ethanol metabolism manipulation, mevalonate production of 1.70 g/L from 200 mM ethanol (about 10 g/L) was enhanced by about 3.8 times to 4.07 g/L and reproducibility of mevalonate production was remarkably enhanced. Third, manipulations to reduce fatty acid biosynthesis and optimize pH by batch fermentation was revealed to be the metabolic characteristic related in ethanol catabolism and mevalonate production. Finally, a product yield of 0.41 g mevalonate/g ethanol was reached in flask scale culture and 0.32 g mevalonate/g ethanol in batch fermentation. This research is expected to open new possibilities for further studies on the production of terpenoids using microorganisms that have not yet reached the stage of mass production.

## Methods

### Heterologous gene expression in *Pseudomonas putida* KT2440

The strains, plasmids, and primers used in this study are listed in Table 1 and Additional file 1 [24–27]. *P. putida*’s two plasmid expression system with different *ori* (origin of replication) was established in this study. pSGP10, *E. coli*–*Pseudomonas* shuttle expression vector, was constructed by insertion of *lacI *+ P_*trc*_ fragment from pTrc99A vector and ColE1 *E. coli ori* + *Tet*^*R*^ (Tetracycline resistance marker) fragment from pCM184 vector into the restriction enzyme (*Nde*I, *Hin*dIII: Takara, Japan) digested pUCP19 vector. pAWP89-0, a broad host-range expression vector was constructed by assembly of P_*lac*_ fragment from pUC19, multi-cloning site (MCS) fragment from MEV vector and the other parts of pAWP89 vector. The *mvaE* (Acetyl-CoA acetyltransferase/Hydroxymethylglutaryl-CoA reductase from *Enterococcus faecalis,* GenBank ID: AAO81155.1)*, mvaS* (Hydroxymethylglutaryl-CoA synthase from *Enterococcus faecalis*, GenBank ID: AAO81154.1), *atoB* (Acetyl-CoA acetyltransferase from *Escherichia coli*, GenBank ID: 946727) for mevalonate production, *acs* (Acetyl-CoA synthetase from *Escherichia coli,* GenBank ID: 948572), *eutE* (Putative aldehyde dehydrogenase/ethanolamine utilization protein from *Escherichia coli,* GenBank ID: 946943) for manipulation of ethanol catabolism, and the *nphT7* (Acetyl-CoA:malonyl-CoA acyltransferase from *Streptomyces* sp. CL190, GenBank ID: AB540131.1) for reducing fatty acid biosynthesis, were codon-optimized for *P. putida* KT2440 and synthesized by Bioneer Co. (Daejeon, Korea). All codon-optimized nucleotide sequences are listed in Additional file 2.

**Table 1:** Bacterial strains and plasmids used in this study

Strain, plasmid	Genotype or properties	Source
Strains		
*P. putida* KT2440	Wild-Type	ATCC
*E. coli* DH10B	*F-endA1recA1 galE15 galK16 nupG rpsL ΔlacX74 Φ80lacZΔM15 araD139Δ(ara,leu)7697 mcrA Δ(mrr-hsdRMS-mcrBC) λ-T1R*	RBC
ELPP000	*P. putida* KT2440 harboring pSGP10, pAWP89-0	This study
ELPP010	*P. putida* KT2440 harboring pSGP11, pAWP89-0	This study
ELPP0dT0	*P. putida* KT2440 harboring pSGP1dT, pAWP89-0	This study
ELPP1dT0	*P. putida* KT2440 *ΔendA ΔendX* harboring pSGP1dT, pAWP89-0	This study
ELPP110	*P. putida* KT2440 *ΔendA ΔendX* harboring pSGP11, pAWP89-0	This study
ELPP111	*P. putida* KT2440 *ΔendA ΔendX* harboring pSGP11, pAWP89-1	This study
ELPP211	*P. putida* KT2440 *ΔendA ΔendX ΔqedH-I ΔqedH-II* harboring pSGP11, pAWP89-1	This study
ELPP212	*P. putida* KT2440 *ΔendA ΔendX ΔqedH-I ΔqedH-II* harboring pSGP11, pAWP89-2	This study
ELPP213	*P. putida* KT2440 *ΔendA ΔendX ΔqedH-I ΔqedH-II* harboring pSGP11, pAWP89-3	This study
ELPP221	*P. putida* KT2440 *ΔendA ΔendX ΔqedH-I ΔqedH-II* harboring pSGP12, pAWP89-1	This study
ELPP311	*P. putida* KT2440 *ΔendA ΔendX ΔqedH-I ΔqedH-II* *ΔphaG* harboring pSGP11, pAWP89-1	This study
Plasmids		
pUCP19	*E. coli–Pseudomonas* Shuttle Expression Vector, P_*lac*_, *Amp*^*R*^	ATCC
pUC19	*E. coli* Expression Vector, *lacI*, P_*lac*_, *Amp*^*R*^	Takara
pTrc99A	*E. coli* Expression Vector, *lacI*, P_*trc*_, *Amp*^*R*^	[24]
pCM184	Allelic Exchange Vector, *Amp*^*R*^, *Tet*^*R*^, *loxP-Kan*^*R*^*-loxP*	[25]
pAWP89	Broad Host Range Expression Vector, P_*tac*_-dTomato, *Kan*^*R*^	[26]
pK19*mobsacB*	Allelic Exchange Vector, *sacB*, *Kan*^*R*^	[27]
pK19*mobsacB* (*::lacI*)	pK19*mobsacB*-*lacI* (derived from pTrc99A)	This Study
pSGP10	*E. coli-Pseudomonas* Shuttle Expression Vector (pUCP19 derived), *lacI* + P_*trc*_ derived from pTrc99A, *Tet*^*R*^ derived from pCM184	This Study
pSGP1dT	pSGP10-dTomato (derived from pAWP89)	This Study
pSGP11	pSGP10-*mvaE*_opti-*mvaS*_opti-*atoB*_opti	This Study
pSGP12	pSGP10-*mvaE*_opti-*mvaS*_opti-*atoB*_opti-*nphT7*_opti	This Study
pAWP89-0	Broad Host Range Expression Vector (pAWP89 derived), P_*lac*_ drived from pUC19, MCS from MEV^a^ Vector	This Study
pAWP89-1	pAWP89-0-*acs*_opti	This Study
pAWP89-2	pAWP89-0-*eutE*_opti	This Study
pAWP89-3	pAWP89-0-*acs*_opti-*eutE*_opti	This Study

^a^Dedicated by professor SEON-WON Kim in Gyeongsang National University

All fragments were amplified by polymerase chain reaction (PCR) from appropriate templates and primers using PrimeSTAR GXL DNA Polymerase (Takara Bio, Shiga, Japan). The PCR conditions were as follows: pre-denaturation 98 °C 5 min, denaturation 98 °C for 10 s—annealing 60 °C 15 s—elongation 68 °C 1 min for 30 cycles, post-elongation 68 °C for 7 min. The PCR products were assembled into the appropriate vector, double restriction enzyme (Takara Bio, Shiga, Japan) digested or linearized by PCR, using Gibson Assembly Master Mix (New England Biolabs, Ipswich, MA, USA). After assembly reaction, the reactants were transformed into *Escherichia coli* DH10B using KCM mediated chemical transformation method [20] and confirmed by restriction enzyme digestion and nucleotide sequencing. The confirmed plasmids were transformed into *P. putida* using previously established electroporation methods [21]. For antibiotics selection, the transformants were spread into appropriate concentration of tetracycline (20 μg/mL for *E. coli* and 30 μg/mL for *P. putida*) and kanamycin (15 μg/mL for *E. coli* and 75 μg/mL for *P. putida*) containing Luria–Bertani (LB) agar plate comprised of 10 g/L of tryptone, 5 g/L of yeast extract, 10 g/L of NaCl, and 10 g/L of agar (Duchefa Biochemie, Haarlem, The Netherlands).

### Manipulation of chromosomal DNA in *Pseudomonas putida* KT2440

*endA* (Endonuclease I, GenBank ID: 1047019) and *endX* (Extracellular DNA endonuclease, GenBank ID: 1045620) for genetic stability improvement, *qedH*-*I* (Quinoprotein ethanol dehydrogenase, GenBank ID: 1046117) and *qedH*-*II* (Quinoprotein ethanol dehydrogenase, GenBank ID: 1046129) for manipulation of ethanol catabolism, and *phaG* ((R)-3-hydroxydecanoyl-ACP:CoA transacylase, GenBank ID: 1046114) for reduction of fatty acid biosynthesis, were deleted from *P. putida* KT2440 chromosomal DNA by *sacB* mediated markerless deletion method, in consecutive order.

*lacI* inserted pK19*mobsacB* vector was constructed for *sacB* mediated markerless deletion. About 500–2000 bp of upstream and downstream region of the target gene was assembled into the *lacI* inserted pK19*mobsacB* vector and then transformed into *P. putida* KT2440 and followed by the selected of plasmid integrated colony by kanamycin (75 μg/mL). To confirm the plasmid integration, first colony PCR was performed with the appropriate primers to amplify the region between the prototype chromosome and integrated plasmid. The confirmed colonies were spread into 12.5% sucrose containing LB agar plate for *sacB* mediated intra-chromosomal recombination, and counter selection in order to distinguish the plasmid integrated colonies, which was performed by replica plating method between LB and LB kanamycin (75 μg/mL) plate. Finally, the deleted mutants were selected by second colony PCR and amplified fragment size comparison using appropriate primers.

### Media and culture conditions

All recombinants were stored at − 80 °C in 25% glycerol stock and inoculated into 3 mL of 30 μg/mL tetracycline and 75 μg/mL kanamycin containing LB broth comprised of 10 g/L of tryptone, 5 g/L of yeast extract, and 10 g/L of NaCl (Duchefa Biochemie, Haarlem, The Netherlands) in a round bottom tube. Seed culture incubated for 14 h at 30 °C and 230 rpm of agitation speed.

Seed culture was inoculated (2% v/v) into 50–100 mL of modified M9 minimal media [22] with 12 g/L of dextrose (BD, Franklin Lakes, NJ, USA) in 250–500 mL bottom-baffled flask and incubated for 24 h at 30 °C and 230 rpm to adapt to M9 minimal media. Adaptation culture was performed to adapt to minimal media prior to inoculation of main culture and to adjust the initial concentration of cells in main culture. The modified M9 minimal medium consisted of K_2_HPO_4_ 16.0 g/L, KH_2_PO_4_ 8 g/L, (NH_4_)_2_SO_4_ 4.7 g/L, NaCl 0.5 g/L, MgSO_4_·7H_2_O 0.12 g/L, FeSO_4_·7H_2_O 6 mg/L, CaCO_3_ 2.7 mg/L, ZnSO_4_·H_2_O 2.0 mg/L, MnSO_4_·H_2_O 1.16 mg/L, CuSO_4_·5H_2_O 0.33 mg/L, CoSO_4_·7H_2_O 0.37 mg/L, H_3_BO_3_ 0.08 mg/L, and HCl 0.01 mL/L (Sigma Aldrich, St. Louis, MO, USA).

After adaptation, M9 adapted culture was inoculated (initial OD_600_ 1.0) into 50 mL of modified M9 minimal media with 10 g/L of ethanol (Merck, Darmstadt, Germany) and 1 g/L of dextrose in 250 mL bottom-baffled flask and incubated for 27 h at 30 °C and 230 rpm. 0.1 mM Isopropyl β-d-1-thiogalactopyranoside (IPTG) induction was performed at the start of the culture. All strains were cultured under the above conditions and in triplet for reproducibility confirmation. Batch fermentation was inoculated from adaptation culture (initial OD_600_ 1.0) in the same manner as flask scale culture and performed in 2.5 L lab-scale fermenter at 1 L working volume. The carbon substrate was increased to 300 mM ethanol and physiological conditions (30 °C, 400 rpm, pH 7.0 and 0.5 vvm aeration) was controlled (in case of pH, by 5 N sodium hydroxide solution). In the case of pH 6.75 and 6.5 culture, only the composition of potassium phosphate buffer was modified (pH 6.75: K_2_HPO_4_ 12.1 g/L, KH_2_PO_4_ 11.0 g/L, pH 6.5: K_2_HPO_4_ 8.2 g/L, KH_2_PO_4_ 14.0 g/L).

### Analytical methods

The optical density (OD) of the cell was assayed with a spectrophotometer (Jenway, Stone, UK) at 600 nm at an appropriate dilution. Samples were withdrawn periodically and then centrifuged at 12,470×*g* for 10 min. The amount of the carbon sources, acetate, and mevalonate in the supernatants were measured on a high-performance liquid chromatography (Shimazu, Kyoto, Japan) system using a Hi-Plex H column (300 × 7.7 mm; Agilent, Santa Clara, CA, USA). Sulfuric acid solution (0.01 N) was used as the mobile phase. The temperature of the column was 40 °C, and the flow rate was 0.6 mL/min. All solutions were filtered through a 0.2 μm polyvinylidene fluoride (PVDF) membrane before analysis.

dTomato gene coding fluorescence protein inserted was into the pSGP10 vector and transformed into *P. putida* KT2440 wild-type and the mutant was studies for genetic stability analysis. Flask culture was performed in 50 mL of modified M9 minimal media with 12 g/L of dextrose. After OD_600_ 0.6 inoculation from adaptation culture, 0.1 mM IPTG induction was performed except for control sample. After 24 h and 48 h from IPTG induction, 1 mL of samples were withdrawn and centrifuged at 12,470×*g* for 5 min at 4 °C. After centrifugation, cells were washed with phosphate buffer saline (PBS; Sigma Aldrich, St. Louis, MO, USA) and resuspended with PBS, then subdivided into 100 μL aliquot at 96 well microplate (Black, Clear Bottom). Fluorescence was analyzed by fluorescence microplate leader (Thermo scientific, Waltham, MA, USA) at excitation wavelength 544 nm and emission wavelength 590 nm.

To quantify the intracellular fatty acid contents, modified fluorometric quantification of neutral lipids using Nile red was performed. Organic solvent and emission wavelength were altered to acetone and 590 nm from previous method [23]. In this study, calibration curve between the fluorescence intensity and fatty acid contents was not measured because only the relative content difference was recorded.

## Supplementary information

**Additional file 1: Table S1.** PCR primers used in this study.

**Additional file 2.** Codon optimized nucleotide sequences used in this study.

## Data Availability

Not applicable.

## Abbreviations

CoA: coenzyme A
PQQ: pyrroloquinoline quinone
NAD: nicotinamide adenine dinucleotide
NADP: nicotinamide adenine dinucleotide phosphate
NADH: nicotinamide adenine dinucleotide, reduced form
NADPH: nicotinamide adenine dinucleotide phosphate, reduced form
ATP: adenosine triphosphate
MEP: 2-C-methyl-d-erythritol 4-phosphate
DOXP: 1-deoxy-d-xylulose 5-phosphate
DMAPP: dimethylallyl pyrophosphate
IPP: isopentenyl pyrophosphate
PHA: polyhydroxyalkanoate
DNA: DeoxyriboNucleic Acid
OD_600_: optical density measured at 600 nm
TCA cycle: tricarboxylic acid cycle
*ori*: origin of replication
PCR: polymerase chain reaction
LB: Luria–Bertani
PVDF: polyvinylidene fluoride
IPTG: isopropyl β-d-1-thiogalactopyranoside
