# Supplementary material for: Mevalonate production from ethanol by direct conversion through acetyl-CoA using recombinant Pseudomonas putida, a novel biocatalyst for terpenoid production
Source: Microb Cell Fact. 2019 Oct 10;18:168. doi: 10.1186/s12934-019-1213-y (PMC6786281; doi:10.1186/s12934-019-1213-y)
Supplement: Supplementary file 1 — Additional file 1: Table S1. PCR primers used in this study. [file 12934_2019_1213_MOESM1_ESM.docx]

**Table S1. PCR Primers used in this study**

| Primer | Sequence |
| --- | --- |
| F-*lacI* (pK19*mobsacB*) | **TGCATGCCTGCAGGTCGAC**TCTAGAGACACCATCGAATGGTGC |
| F-*lacI* (pK19*mobsacB*) | **AAAACGACGGCCAGT**GAATTCTCACTGCCCGCTTTCC |
| F-*endA-*upstream | **CTATGACCATGATTACGCC**AAGCTTTGCTGCTCTTGAAATGAACC |
| R-*endA-*upstream | **TTTGAAACGG**GGGGAAAACATATTTCAGGTTG |
| F-*endA-*downstream | **TGTTTTCCCC**CCGTTTCAAAGGCTGCG |
| R-*endA-*downstream | **TGCACCATTCGATGGTGTC**TCTAGAATGAAGAAGCGAATCGTCCT |
| F-*endX*-upstream | **CTATGACCATGATTACGCC**AAGCTTGTGCTTCCCCCTCAGGG |
| R-*endX*-upstream | **GGCCTGAGGA**GCGCAGTCAATCTTCCTTCG |
| F-*endX*-downstream | **TTGACTGCGC**TCCTCAGGCCAGCGTTTG |
| R-*endX*-downstream | **TGCACCATTCGATGGTGTC**TCTAGAACCAGTAAAAGTGGCGCCG |
| F-*qedH-I*-upstream | **CTATGACCATGATTACGCC**AAGCTTGTGGCCATGAACTGGCG |
| R-*qedH-I*-upstream | **CCGCTGCAGG**GGTTGCAGTTCCCAGTGGA |
| F-*qedH-I*-downstream | **AACTGCAACC**CCTGCAGCGGGGAGC |
| R-*qedH-I*-downstream | **TGCACCATTCGATGGTGTC**TCTAGAATGAATATCGTGTTGGTCGATGAC |
| F-*qedH-II*-upstream | **CTATGACCATGATTACGCC**AAGCTTGTGGCCATGAACTGGCG |
| R-*qedH-II*-upstream | **TAGGCAGGCG**GACGGCTACCTTTGGTTTTTTTG |
| F-*qedH-II*-downstream | **GGTAGCCGTC**CGCCTGCCTACTGCCG |
| R-*qedH-II*-downstream | **TGCACCATTCGATGGTGTC**TCTAGATTAGAAGAAGCCCAGCGGAT |
| F-*phaG*-upstream | **CTATGACCATGATTACGCC**AAGCTTGTGTCTGCAGTGAAACCCG |
| R-*phaG*-upstream | **GCCGAGCCGC**GTCATCGACTCCTGGCGC |
| F-*phaG*-downstream | **AGTCGATGAC**GCGGCTCGGCGCC |
| R-*phaG*-downstream | **TGCACCATTCGATGGTGTC**TCTAGACTAACCCTGTTCGGTCACTTG |
| F-Confirm1-upstream | (Universal) CGATTCATTAATGCAGCTGGC |
| R-Confirm1-downstream | (Universal) TCCACTTTTTCCCGCGTTTTC |
| R-Confirm1-upstream | (*endA*) GGCACGATGTGTTCCCAC |
| F-Confirm1-downstream | (*endA*) AAGCCAGAGGCCAAACCAA |
| R-Confirm1-upstream | (*endX*) TTTACAGCCGCAATAAAACTCGG |
| F-Confirm1-downstream | (*endX*) AAGCCTGGGAGCGGCAA |
| R-Confirm1-upstream | (*qedH-I*) TGGCCGCTGTTGCCA |
| F-Confirm1-downstream | (*qedH-I*) CGTGGGCTACGGCGG |
| R-Confirm1-upstream | (*qedH-II*) GCATCGAGCATGCGCAAG |
| F-Confirm1-downstream | (*qedH-II*) ATCTCCAGGTCCGCCGC |
| R-Confirm1-upstream | (*phaG*) GCCGTGGTGGCCAGC |
| F-Confirm1-downstream | (*phaG*) CCCGCAATGTCATGCTGG |
| F-Confirm2-*endA* | TGCTGCTCTTGAAATGAACC |
| R-Confirm2-*endA* | ATGAAGAAGCGAATCGTCCT |
| F-Confirm2-*endX* | GCAACGTCACCGACACC |
| R-Confirm2-*endX*-R | AGCTCTGCGGTGGAGC |
| F-Confirm2-*qedH-I* | GCATCGAGCATGCGCAAG |
| R-Confirm2-*qedH-I* | ATCTCCAGGTCCGCCGC |
| F-Confirm2-*qedH-II* | CACCGCCTGAGGTTGCT |
| R-Confirm2-*qedH-II* | AGCCACGGTGCCTTCG |
| F-Confirm2-*phaG* | TCCGCAACACCGTACCG |
| R-Confirm2-*phaG* | GCCGATCAGGATCGGCC |
| F-*lacI*+P*_trc_* (pSGP10) | **GTGCGGTATTTCACACCG**CATATGGGCTTCACCTTCAACCCAACAC |
| R-*lacI*+P*_trc_* (pSGP10) | **CGATGGTGT**CGAGCGTCAGACCCCGTAG |
| F-ori+*Tet^R^* (pSGP10) | **TCTGACGCTC**GACACCATCGAATGGTGCA |
| R-ori+*Tet^R^* (pSGP10) | **GACCTGCAGGCATGC**AAGCTTCATGGTCTGTTTCCTGTGTGAA |
| F-dTomato | **TGCATGCCTGCAGGTCGAC**TCTAGAATGGTGAGCAAGGGCGAG |
| R-dTomato | **TGAATTCGAGCTCGGTA**CCCGGGCTACTTGTACAGCTCGTCCATG |
| F-*mvaE*_opti | **AAGCTTGCATGCCTGCAGGTCGAC**TCTAGAATTTAAGGAGAACTTTATATGAAGACCGT |
| R-*mvaE*_opti | **AAAGTGTCTAGGA**TTATTGCTTACGCAAATCGTTCA |
| F-*mvaS*_opti | **GCGTAAGCAATAA**TCCTAGACACTTTCACCATAAGGA |
| R-*mvaS*_opti | **TACCCAGCGGACT**TTAGTTGCGATAGCTGCGC |
| F-*atoB*_opti | **CTATCGCAACTAA**AGTCCGCTGGGTAGACTAAGG |
| R-*atoB*_opti | **GCCAGTGAATTCGAGCTCGGTA**CCCGGGTCAGTTCAGGCGCTCGATC |
| R-*atoB*_opti (pSGP12) | **CTACAGAACTTAA**TCAGTTCAGGCGCTCGATC |
| F-*nphT7*_opti | **GCGCCTGAACTGA**TTAAGTTCTGTAGGGCCGAGAC |
| R-*nphT7*_opti | **GCCAGTGAATTCGAGCTCGGTA**CCCGGGTCACCACTCGATCAGCGC |
| F-*acs*_opti | **TTTCACACAGGAAACAGC**GGGCCCCTGAGAATAGCCCTCAACTACGT |
| F-*acs*_opti (pAWP89-3) | **CATCGTGTGA**CTGAGAATAGCCCTCAACTACGT |
| R-*acs*_opti | **GATAGTCTAGAAGGTACCA**GAATTCTCAGCTCGGCATGGCG |
| F-*eutE*_opti | **TTTCACACAGGAAACAGC**GGGCCCAGAAAGACAAGAGATAAGGAGGT |
| R-*eutE*_opti | **GATAGTCTAGAAGGTACCA**GAATTCTCACACGATGCGGAAGGC |
| R-*eutE*_opti (pAWP89-3) | **CTATTCTCAG**TCACACGATGCGGAAGGC |

^a^Bold letters indicate homologous sequence for assembly reaction, underlined letters indicate restriction site.
