# Supplementary material for: Mevalonate production from ethanol by direct conversion through acetyl-CoA using recombinant Pseudomonas putida, a novel biocatalyst for terpenoid production
Source: Microb Cell Fact. 2019 Oct 10;18:168. doi: 10.1186/s12934-019-1213-y (PMC6786281; doi:10.1186/s12934-019-1213-y)
Supplement: Supplementary file 2 — Additional file 2. Codon optimized nucleotide sequences used in this study. [file 12934_2019_1213_MOESM2_ESM.docx]

*mvaE*_opti (Acetyl-CoA acetyltransferase / Hydroxymethylglutaryl-CoA reductase) from *Enterococcus faecalis*

ATTTAAGGAGAACTTTATATGAAGACCGTGGTCATCATCGATGCCCTGCGCACCCCGATCGGCAAATACAAAGGCTCGCTCAGCCAGGTCTCGGCCGTGGACCTGGGCACCCACGTGACCACCCAGTTGCTGAAGCGCCACAGCACGATCAGCGAAGAGATCGACCAGGTTATCTTTGGGAACGTGCTGCAGGCGGGTAACGGCCAGAACCCCGCGCGTCAGATCGCCATCAACTCGGGGCTGTCGCATGAAATCCCGGCCATGACCGTGAATGAGGTCTGCGGCTCCGGCATGAAGGCGGTCATCCTGGCTAAGCAGCTGATCCAGCTGGGCGAAGCCGAGGTGCTCATCGCCGGCGGCATCGAAAACATGAGCCAGGCCCCGAAGCTGCAGCGGTTCAACTATGAAACTGAGAGTTACGACGCCCCGTTCAGCAGCATGATGTACGACGGCCTGACGGATGCGTTCAGCGGCCAGGCAATGGGCCTGACCGCCGAGAACGTGGCCGAGAAGTACCACGTGACCCGCGAGGAACAGGACCAGTTCAGCGTGCACTCCCAGCTGAAGGCCGCACAGGCCCAAGCCGAAGGCATCTTCGCCGACGAGATTGCGCCCCTGGAAGTTAGCGGCACGCTCGTGGAGAAGGATGAGGGCATCCGTCCAAACTCGAGCGTGGAGAAACTGGGCACCCTGAAGACCGTGTTCAAGGAGGACGGCACCGTCACTGCCGGGAACGCGTCCACCATCAACGACGGGGCCTCGGCCCTGATTATCGCTAGTCAGGAATATGCCGAGGCCCATGGCCTGCCGTACTTGGCGATCATCCGCGACTCGGTGGAGGTCGGCATCGACCCGGCGTACATGGGCATCAGCCCGATCAAAGCCATCCAGAAGCTCTTGGCCCGCAACCAACTGACGACCGAAGAGATCGATCTGTACGAAATCAATGAGGCCTTCGCTGCCACCAGCATCGTCGTACAGCGCGAACTGGCGCTGCCGGAGGAAAAGGTGAACATCTACGGTGGTGGCATCAGCCTGGGTCACGCGATCGGCGCAACCGGCGCCCGCCTGCTGACCAGCCTTTCCTACCAGCTGAACCAAAAGGAGAAAAAGTATGGTGTCGCCAGCCTCTGCATTGGGGGCGGCCTGGGCCTTGCCATGCTGTTGGAGCGTCCGCAGCAGAAAAAGAATTCGCGCTTTTACCAGATGTCCCCGGAGGAGCGCCTGGCCTCCCTGCTGAACGAAGGCCAGATCTCGGCGGATACCAAGAAGGAGTTCGAGAACACGGCTCTGAGCTCGCAGATCGCCAACCACATGATTGAGAACCAGATCAGCGAGACCGAGGTTCCCATGGGCGTCGGCCTGCACCTGACCGTGGATGAGACCGACTACCTGGTGCCGATGGCCACCGAGGAGCCGAGTGTCATCGCCGCACTGAGCAACGGCGCCAAGATCGCGCAGGGCTTCAAAACCGTGAACCAGCAGCGCCTGATGCGGGGCCAGATCGTGTTCTACGACGTGGCGGACGCCGAAAGCCTGATCGACGAGCTGCAGGTGCGCGAGACCGAGATCTTCCAACAGGCCGAGCTCAGCTACCCGAGCATCGTAAAGCGCGGCGGGGGCCTGCGCGACCTGCAATACCGGGCGTTCGACGAGTCTTTCGTTAGTGTGGATTTCCTGGTCGACGTCAAGGACGCGATGGGTGCCAACATCGTGAATGCAATGTTGGAAGGTGTCGCCGAGCTGTTCCGCGAATGGTTCGCCGAACAAAAAATCCTGTTCAGCATCCTGTCCAACTACGCCACCGAGTCGGTCGTGACGATGAAGACGGCGATCCCGGTAAGCCGCCTCAGCAAAGGTAGCAACGGCCGCGAGATCGCAGAAAAGATCGTGCTGGCCTCACGCTATGCCAGCCTGGACCCCTACCGCGCCGTGACCCATAACAAGGGCATCATGAACGGTATTGAGGCGGTAGTGCTGGCGACCGGCAATGACACCCGTGCCGTGAGCGCATCGTGCCACGCATTTGCCGTCAAGGAAGGCCGGTACCAGGGCTTGACCTCCTGGACCCTGGACGGCGAACAGCTGATTGGCGAAATCAGCGTGCCGCTCGCGCTTGCCACCGTGGGCGGGGCCACTAAGGTGCTGCCGAAAAGCCAGGCCGCCGCTGACCTGCTGGCGGTTACCGATGCAAAAGAGCTGTCGCGCGTCGTCGCCGCTGTGGGGCTGGCCCAAAACTTGGCCGCCCTGCGCGCGCTCGTGTCGGAGGGCATCCAGAAGGGCCACATGGCTCTGCAGGCCCGTAGCCTGGCCATGACAGTGGGGGCCACCGGCAAGGAGGTCGAAGCGGTAGCGCAGCAGCTGAAGCGCCAGAAAACCATGAACCAGGACCGCGCCCTGGCTATCCTGAACGATTTGCGTAAGCAATAA

*mvaS*_opti (Hydroxymethylglutaryl-CoA synthase) from *Enterococcus faecalis*

TCCTAGACACTTTCACCATAAGGAAATATTTTAATGACCATCGGCATCGATAAAATCAGCTTCTTCGTGCCCCCGTATTACATCGACATGACTGCTTTGGCCGAAGCACGCAACGTCGATCCAGGGAAATTTCACATCGGCATCGGCCAGGACCAGATGGCGGTAAACCCGATCAGCCAAGACATCGTCACCTTCGCCGCCAACGCCGCCGAGGCGATCCTCACCAAGGAAGATAAGGAGGCTATTGACATGGTGATCGTGGGGACCGAGAGCAGCATCGACGAGTCCAAGGCCGCCGCCGTGGTGCTGCACCGCCTGATGGGCATTCAGCCGTTCGCGCGCTCGTTCGAGATCAAGGAAGCCTGCTACGGCGCAACGGCAGGCCTGCAGCTGGCCAAGAACCACGTTGCGCTGCATCCGGACAAAAAGGTGCTGGTCGTGGCGGCTGATATCGCGAAGTACGGTCTGAACAGCGGCGGCGAACCCACCCAGGGCGCGGGCGCCGTGGCCATGCTCGTGGCCTCGGAGCCGCGGATTCTGGCCCTGAAGGAAGACAATGTCATGTTGACCCAGGACATCTACGACTTCTGGCGTCCTACCGGCCACCCGTACCCCATGGTCGACGGCCCGCTGAGCAACGAGACCTACATCCAGTCCTTCGCACAGGTGTGGGACGAGCACAAGAAGCGCACCGGCCTCGATTTCGCCGACTATGATGCGCTGGCCTTTCACATCCCGTACACCAAGATGGGCAAAAAGGCGCTGCTGGCGAAGATCAGCGATCAGACGGAGGCCGAGCAAGAGCGGATCCTGGCCCGTTACGAGGAGTCGATCATCTACAGCCGCCGCGTCGGCAATCTGTACACCGGCAGCCTGTACCTGGGTCTGATTTCGCTGCTGGAGAACGCTACCACCCTGACCGCGGGGAACCAGATCGGCCTGTTCAGCTACGGTAGTGGCGCCGTGGCCGAGTTCTTCACCGGCGAGCTGGTGGCCGGCTACCAGAACCATCTGCAGAAGGAGACGCACCTCGCCCTGCTGGACAATCGCACCGAGCTGTCGATCGCCGAGTACGAGGCCATGTTCGCGGAGACCCTGGACACGGACATCGACCAGACCCTGGAGGACGAGCTGAAATACAGCATCTCCGCCATCAACAACACCGTGCGCAGCTATCGCAACTAA

*atoB*_opti (Acetyl-CoA acetyltransferase) from *Escherichia coli* MG1655

AGTCCGCTGGGTAGACTAAGGAGGTTATAGTATGAAGAACTGCGTGATCGTGAGCGCCGTGCGCACCGCCATCGGCAGCTTCAACGGCAGCCTGGCCAGCACCAGCGCCATCGATCTGGGTGCCACCGTGATCAAAGCCGCCATCGAGCGTGCCAAGATCGACAGCCAGCACGTGGACGAGGTGATCATGGGCAACGTGCTGCAAGCCGGTCTGGGTCAGAACCCAGCGCGTCAGGCCCTGCTGAAAAGCGGTCTGGCCGAAACCGTGTGCGGCTTCACCGTGAACAAGGTGTGCGGCAGCGGTCTGAAGTCGGTGGCCCTGGCCGCGCAAGCCATCCAAGCGGGTCAAGCCCAGAGCATCGTGGCCGGTGGCATGGAAAACATGTCGCTGGCCCCATACCTGCTGGACGCCAAAGCGCGTAGCGGCTACCGCCTGGGTGACGGCCAGGTGTACGACGTGATCCTGCGCGACGGCCTGATGTGCGCGACCCACGGCTACCACATGGGCATCACCGCCGAGAACGTGGCCAAAGAGTACGGCATCACCCGCGAGATGCAGGACGAGCTGGCCCTGCACAGCCAGCGTAAAGCCGCCGCCGCGATCGAGAGCGGTGCCTTCACCGCGGAAATCGTGCCGGTGAACGTGGTGACCCGCAAGAAAACCTTCGTGTTCAGCCAGGACGAGTTCCCGAAGGCCAACAGCACCGCGGAGGCCCTGGGTGCCCTGCGTCCAGCCTTCGATAAAGCCGGCACCGTGACCGCCGGTAACGCCAGCGGCATCAACGACGGTGCCGCCGCGCTGGTCATCATGGAAGAAAGCGCGGCCCTGGCGGCCGGTCTGACCCCACTGGCGCGTATCAAGAGCTACGCGAGCGGTGGTGTCCCGCCAGCGCTGATGGGTATGGGTCCAGTGCCAGCCACCCAGAAGGCCCTGCAACTGGCGGGTCTGCAGCTGGCCGACATCGACCTGATCGAGGCCAACGAGGCCTTCGCCGCGCAGTTCCTGGCCGTGGGCAAGAACCTGGGCTTCGACAGCGAGAAGGTGAACGTCAACGGTGGTGCGATCGCCCTGGGCCATCCGATCGGTGCCAGCGGTGCCCGTATCCTGGTGACCCTGCTGCATGCCATGCAAGCCCGTGACAAGACCCTGGGCCTGGCGACCCTGTGCATCGGTGGCGGTCAGGGTATCGCCATGGTGATCGAGCGCCTGAACTGA

*acs_*opti (Acetyl-CoA synthetase) from *Escherichia coli* MG1655

CTGAGAATAGCCCTCAACTACGTAAGGAGGTATTTATGAGCCAGATCCACAAGCACACCATCCCAGCCAACATCGCCGACCGCTGCCTGATCAACCCGCAGCAGTACGAGGCCATGTACCAGCAGAGCATCAACGTGCCGGACACCTTCTGGGGTGAGCAGGGCAAGATCCTGGACTGGATCAAGCCGTACCAGAAGGTCAAGAACACCAGCTTCGCCCCAGGCAACGTGAGCATCAAGTGGTACGAGGACGGCACCCTGAACCTGGCCGCCAACTGCCTGGACCGCCATCTGCAAGAGAACGGCGACCGCACCGCCATCATCTGGGAAGGCGACGACGCCAGCCAGAGCAAGCACATCAGCTACAAAGAACTGCACCGCGACGTGTGCCGCTTCGCGAACACCCTGCTGGAACTGGGCATCAAGAAAGGCGACGTGGTGGCCATCTACATGCCGATGGTGCCGGAAGCCGCCGTGGCCATGCTGGCCTGCGCGCGTATCGGTGCCGTGCACAGCGTGATCTTCGGTGGCTTCAGCCCAGAGGCCGTGGCCGGTCGCATCATCGACAGCAACAGCCGTCTGGTCATCACCAGCGACGAGGGCGTGCGTGCCGGTCGTAGCATCCCGCTGAAGAAGAACGTCGACGACGCGCTGAAAAACCCGAACGTGACCAGCGTGGAACACGTGGTGGTGCTGAAACGCACCGGTGGCAAGATCGACTGGCAAGAGGGTCGCGATCTGTGGTGGCACGACCTGGTGGAACAGGCCAGCGACCAGCACCAGGCCGAGGAAATGAACGCGGAGGACCCGCTGTTCATCCTGTACACCAGCGGCAGCACCGGCAAGCCGAAGGGCGTGCTGCATACCACCGGTGGTTACCTGGTGTATGCCGCGCTGACCTTCAAGTACGTGTTCGACTACCATCCGGGTGACATCTACTGGTGCACCGCCGATGTCGGTTGGGTCACCGGTCACAGCTACCTGCTGTACGGTCCGCTGGCGTGCGGTGCGACCACCCTGATGTTCGAAGGCGTGCCGAACTGGCCGACCCCAGCGCGTATGGCCCAGGTGGTGGACAAGCACCAGGTGAACATCCTGTATACCGCGCCAACCGCCATCCGTGCGCTGATGGCCGAAGGCGACAAGGCCATCGAAGGCACCGACCGCAGCAGCCTGCGTATCCTGGGCAGCGTGGGCGAGCCGATCAACCCCGAAGCCTGGGAGTGGTACTGGAAGAAGATCGGCAACGAGAAGTGCCCAGTGGTCGACACCTGGTGGCAGACCGAAACCGGTGGCTTCATGATCACCCCACTGCCAGGTGCCACCGAGCTGAAAGCCGGTAGCGCGACCCGTCCGTTCTTCGGCGTGCAGCCAGCGCTGGTCGACAACGAGGGCAACCCGCTGGAAGGTGCGACCGAGGGCAGCCTGGTGATCACCGACAGCTGGCCAGGCCAAGCGCGTACCCTGTTCGGCGACCACGAGCGCTTCGAGCAGACCTACTTCAGCACCTTCAAGAACATGTACTTCAGCGGTGACGGTGCCCGTCGTGACGAGGATGGCTACTACTGGATCACCGGTCGCGTGGACGACGTCCTGAACGTGAGCGGTCACCGTCTGGGCACCGCGGAGATCGAAAGCGCCCTGGTGGCCCATCCGAAGATCGCCGAAGCCGCGGTGGTGGGCATCCCGCACAACATCAAGGGCCAAGCCATCTACGCCTACGTGACCCTGAACCACGGCGAGGAACCGTCGCCAGAGCTGTACGCCGAGGTGCGCAACTGGGTGCGCAAAGAGATCGGTCCCCTGGCGACCCCAGACGTGCTGCACTGGACCGACAGCCTGCCAAAGACCCGCAGCGGCAAAATCATGCGTCGCATCCTGCGCAAGATCGCGGCCGGTGACACCAGCAACCTGGGCGACACCTCGACCCTGGCCGATCCAGGCGTGGTGGAAAAGCTGCTGGAAGAGAAGCAAGCGATCGCCATGCCGAGCTGA

*eutE_*opti (Putative aldehyde dehydrogenase / ethanolamine utilization protein) from *Escherichia coli* MG1655

AGAAAGACAAGAGATAAGGAGGTATTTTATGAACCAGCAGGACATCGAACAGGTGGTGAAGGCCGTGCTGCTGAAGATGCAGAGCAGCGACACCCCGAGCGCCGCCGTGCATGAGATGGGTGTGTTCGCCAGCCTGGACGACGCCGTGGCCGCCGCCAAGGTGGCCCAGCAAGGTCTGAAGTCGGTGGCCATGCGTCAGCTGGCGATCGCGGCCATCCGCGAAGCCGGTGAGAAGCATGCCCGTGACCTGGCCGAACTGGCCGTGAGCGAAACCGGCATGGGTCGCGTCGAGGACAAGTTCGCCAAGAACGTGGCCCAAGCGCGTGGCACCCCAGGCGTGGAATGCCTGTCGCCACAGGTGCTGACCGGTGACAACGGTCTGACCCTGATCGAGAACGCGCCATGGGGTGTCGTGGCCAGCGTGACCCCAAGCACCAACCCAGCCGCCACCGTGATCAACAACGCCATCAGCCTGATCGCCGCCGGTAACAGCGTGATCTTCGCCCCACATCCAGCCGCGAAGAAAGTGAGCCAGCGTGCGATCACCCTGCTGAACCAGGCCATCGTGGCGGCCGGTGGTCCGGAAAACCTGCTGGTGACCGTGGCGAACCCCGACATCGAAACCGCGCAGCGCCTGTTCAAGTTCCCAGGCATCGGCCTGCTGGTCGTCACCGGTGGCGAAGCCGTGGTGGAAGCCGCGCGTAAGCACACCAACAAGCGCCTGATCGCGGCCGGTGCGGGTAACCCACCAGTGGTGGTGGACGAAACCGCCGACCTGGCGCGTGCCGCGCAGAGCATCGTGAAGGGTGCCAGCTTCGACAACAACATCATCTGCGCCGACGAGAAGGTGCTGATCGTGGTGGACAGCGTGGCCGACGAGCTGATGCGCCTGATGGAAGGCCAGCACGCCGTGAAGCTGACCGCCGAACAGGCCCAGCAGCTGCAGCCGGTCCTGCTGAAAAACATCGACGAGCGTGGCAAGGGCACCGTGAGCCGTGATTGGGTGGGTCGTGATGCCGGTAAGATCGCCGCGGCCATCGGCCTGAAGGTGCCGCAAGAAACCCGTCTGCTGTTCGTGGAAACCACCGCCGAGCATCCGTTCGCCGTGACCGAACTGATGATGCCCGTGCTGCCGGTGGTGCGCGTGGCCAACGTGGCCGATGCGATCGCCCTGGCCGTCAAGCTGGAAGGCGGTTGCCATCACACCGCCGCCATGCACAGCCGCAACATCGAGAACATGAACCAGATGGCCAACGCGATCGACACCAGCATCTTCGTGAAGAACGGTCCGTGCATCGCCGGTCTGGGCCTGGGTGGCGAAGGCTGGACCACCATGACCATCACCACCCCGACCGGTGAGGGCGTGACCAGCGCGCGTACCTTCGTGCGTCTGCGTCGCTGCGTGCTGGTCGACGCCTTCCGCATCGTGTGA

*nphT7*_opti (Acetyl-CoA:malonyl-CoA acyltransferase) from *Streptomyces* sp. CL190

TTAAGTTCTGTAGGGCCGAGACTAAGGAGGTTTTTTATGACCGACGTGCGCTTCCGCATCATCGGCACCGGTGCGTACGTGCCGGAACGCATCGTGAGCAACGACGAGGTGGGTGCCCCAGCCGGTGTGGACGACGATTGGATCACCCGCAAGACCGGCATCCGCCAGCGTCGTTGGGCCGCCGATGATCAGGCCACCTCGGACCTGGCCACCGCCGCCGGTCGTGCCGCGCTGAAAGCCGCCGGTATCACCCCAGAGCAGCTGACCGTGATCGCCGTGGCGACCAGCACCCCAGATCGTCCGCAGCCACCAACCGCCGCCTACGTGCAGCATCACCTGGGTGCCACCGGCACCGCGGCCTTCGACGTGAACGCCGTGTGCAGCGGCACCGTGTTCGCCCTGAGCAGCGTGGCGGGCACCCTGGTGTATCGCGGTGGCTACGCCCTGGTGATCGGTGCCGACCTGTACAGCCGCATCCTGAACCCAGCCGACCGCAAAACCGTGGTGCTGTTCGGCGACGGTGCGGGTGCCATGGTGCTGGGTCCGACCTCGACCGGCACCGGTCCAATCGTGCGTCGCGTGGCCCTGCACACCTTCGGTGGTCTGACCGACCTGATCCGCGTGCCAGCCGGTGGTAGCCGTCAGCCGCTGGATACCGATGGCCTGGATGCGGGTCTGCAGTACTTCGCCATGGACGGTCGCGAGGTGCGTCGCTTCGTGACCGAGCATCTGCCGCAGCTGATCAAGGGCTTCCTGCACGAAGCGGGTGTCGATGCCGCCGACATCAGCCACTTCGTGCCGCACCAAGCCAACGGCGTGATGCTGGACGAGGTGTTCGGTGAGCTGCATCTGCCACGTGCCACCATGCACCGTACCGTGGAAACCTACGGCAACACCGGTGCGGCGAGCATCCCCATCACCATGGATGCGGCCGTGCGTGCCGGTAGCTTCCGTCCGGGTGAACTGGTGCTGCTGGCCGGTTTCGGTGGCGGTATGGCCGCCAGCTTCGCGCTGATCGAGTGGTGA
